# Supplementary material for: Dickkopf1 induces enteric neurogenesis and gliogenesis in vitro if apoptosis is evaded
Source: Commun Biol. 2023 Aug 2;6:808. doi: 10.1038/s42003-023-05072-x (PMC10397193; doi:10.1038/s42003-023-05072-x)
Supplement: Supplementary file 2 — Supplementary Information [file 42003_2023_5072_MOESM2_ESM.pdf]

Supplementary information

Dickkopf1 induces enteric neurogenesis and gliogenesis *in vitro* if apoptosis is evaded

Melanie Scharr[1], Simon Scherer[2], Bernhard Hirt[1], Peter H. Neckel[1*]

[1] Institute of Clinical Anatomy and Cell Analysis, University of Tübingen, Tübingen, Germany

[2] Department of Pediatric Surgery, University Children's Hospital Tübingen, Germany

*** Corresponding author:**
Peter H. Neckel
Institute of Clinical Anatomy and Cell Analysis
Eberhard Karls University Tübingen
Österbergstraße 3
72074 Tübingen, Germany
Phone: +49-7071-29-72169
Fax: +49-7071-29-4014
Email: pneckel@uni-tuebingen.de

Supplementary Tables

Supplementary Table 1: intestinal resectates used in this study

age in months	sex	diagnosis	gut region	experiment
4	female	imperforate anus	transverse colon	Cell culture
24	male	short-gut syndrome	jejunum	Cell culture
6	male	imperforate anus	transverse colon	Cell culture
7	female	imperforate anus	transverse colon	Histology
7	female	imperforate anus	transverse colon	Histology
24	female	imperforate anus	transverse colon	Histology
34	male	obstruction syndrome	jejunum	Histology
4	male	imperforate anus	ileum	Histology
3	male	imperforate anus	ileum	Histology

Supplementary Table 2: primary and secondary antibodies used in this study

primary antibodies	host	dilution	manufacturer
Active β-catenin	mouse	1:500	Merck Millipore, MA, USA
BrdU	rat	1:100	Bio-Rad, NC, USA
Dkk1	mouse	1:50	Santa Cruz, CA, USA
HuC/D	mouse	1:50	Invitrogen Thermo Fisher Scientific, MA, USA
GAPDH	mouse	1:1000	Calbiochem, Darmstadt, Germany
GFAP	rabbit	1:400	DAKO, Glostrup, Denmark
Ki67	rabbit	1:100	DCS Innovative Diagnostics, Hamburg, Germany
PCNA	mouse	1:1000	Sigma-Aldrich, Taufkirchen, Germany
S100β	rabbit	1:100	Abcam, Cambridge, UK
SOX10	mouse	1:50	Novus Biologicals, CO, USA
**secondary antibodies**	**host**	**dilution**	**manufacturer**
α rabbit 546-conjugated	goat	1:400	Invitrogen Thermo Fisher Scientific, MA, USA
α rat 488-conjugated	goat	1:400	Invitrogen Thermo Fisher Scientific, MA, USA
α mouse 546-conjugated	goat	1:400	Invitrogen Thermo Fisher Scientific, MA, USA
α mouse Cy5-conjugated	goat	1:200	Jackson Immuno Research Laboratories INC., PA, USA
α mouse 546-conjugated	goat	1:400	Invitrogen Thermo Fisher Scientific, MA, USA
α mouse Cy5-conjugated	goat	1:200	Jackson Immuno Research Laboratories INC., PA, USA

Supplementary Table 3: Primer used in this study

primer	primer sequence or TaqMan assay probe no.	primer (bps)	amplicon (bp)
dkk1	fwd 5´-ccgggaactactgcaaaaat-3´	20	127/94
	rev 5`-ccaaggttttcaatgatgctt-3´	21	
dkk2	fwd 5´-ctggtacccgctgcaataat-3´	20	105
	rev 5`-catggttgcgatctctatgc-3´	20	
dkk3	fwd 5´-tcgtgaccagatccagctt-3´	19	87/81
	rev 5`-agccgctgcatgtttgtt-3´	18	
dkk4	fwd 5´-acgaagaaatcacaaagcagtaag-3´	24	83/94
	rev 5`-aaaaatggcgagcacagc-3´	18	
krm1	Mm00459616		74
krm2	Mm01309205		103
lrp5	fwd 5´-catggacatccaagtgctga-3´	20	66
	rev 5`-ttgtcctcctcgcatggt-3´	18	
lrp6	fwd 5´-tcctcgagctctggcact-3´	18	60
	rev 5`-cctccccactcagtccaata-3´	20	
gapdh	fwd 5´-agcttgtcatcaacgggaag-3´	30	62
	rev 5`-tttgatgttagtggggtctcg-3´	21	
hprt	fwd 5´-tcctcctcagaccgctttt-3´	19	90
	rev 5`-cctggttcatcatcgctaatc-3´	21	
tbp	fwd 5`-ggcggtttggctaggttt -3´	18	83
	rev 5`-gggttatcttcacacaccatga -3´	22	
axin2	fwd 5`-aggaaccactcggctgct-3´	18	90
	rev 5`-cagtttctttggctctttgtga-3´	22	
lef1	fwd 5`-tcctgaaatccccaccttc-3´	19	100
	rev 5`-acccgtgatgggataaacag-3´	20	
lgr5	fwd 5`-cttcactcggtgcagtgct-3´	19	72

Supplementary Table 4: HiPlex Target probes used in this study.

**Target probe**	**Channel**
Mus musculus kringle containing transmembrane protein 1 (Kremen1)	Atto647N
Mus musculus kringle containing transmembrane protein 2 (Kremen2)	Atto550
Mus musculus dickkopf homolog 1 (Xenopus laevis) (Dkk1)	Atto647N
Mus musculus dickkopf homolog 2 (Xenopus laevis) (Dkk2)	AF488
Mus musculus dickkopf homolog 3 (Xenopus laevis) (Dkk3)	Atto550
Mus musculus dickkopf homolog 4 (Xenopus laevis) (Dkk4)	Atto647N
Mus musculus low density lipoprotein receptor-related protein 5 (Lrp5)	AF488
Mus musculus low density lipoprotein receptor-related protein 6 (Lrp6)	Atto550

Supplementary Table 5: Expression of mRNAs in the murine intestine.
(-) no expression, (+) low expression, (++) medium expression, (+++) high expression

**Small intestine**					
**Target probe**	submucous plexus	myenteric plexus	tunica muscularis	crypts	villus surface
Dkk1	+	++	++	+	+
Dkk2	+++	+++	++	+	+
Dkk3	+++	+++	++	++	++
Dkk4	+	+	-	-	-
Lrp5	+++	+++	+++	+++	-
Lrp6	+++	+++	+++	+++	-
Krm1	+++	+++	+	++	-
Krm2	+++	+++	+++	++	+++
**Large intestine**					
**Target probe**	submucous plexus	myenteric plexus	tunica muscularis	crypts	epithelial surface
Dkk1	+	+	+	+	+
Dkk2	+++	+++	++	+	+
Dkk3	+++	+++	+++	+++	+++
Dkk4	++	++	+	+	+
Lrp5	+++	+++	++	+++	-
Lrp6	+++	+++	++	+++	-
Krm1	+++	+++	+	++	-
Krm2	+++	+++	++	++	+

Supplementary Table 6: Quantification data of HuC/D and GFAP positive cells in human ENS cultures. Patient 1-3 correspond to the three intestinal resectates used in this study for cell culture experiments (see also Supplementary Table 1).

**HuC/D**	Patient 1	Patient 2	Patient 3
control	218	29	24
DKK1	216	30	23
WNT3A	366	46	43
DKK1+WNT3A	456	54	48
**GFAP**	Patient 1	Patient 2	Patient 3
control	616	260	357
DKK1	476	148	362
WNT3A	896	350	684
DKK1+WNT3A	856	360	551

Supplementary Table 7: Total number of glia marker positive cells ($SOX10^+S100beta^+$ and $S100beta^+$ and $SOX10^+$) in murine ENS cultures for all pharmacological treatments.

**Figure 5B**	**Seeded cells/cm²**	**mean±SD**
control	20.000	15374±782
DKK1	20.000	14477±75
WNT3A	20.000	20109±1330
DKK1+WNT3A	20.000	21979±2714
**Supplementary Figure 11**	**Seeded cells/cm²**	**mean±SD**
control	5.000	6103±3141
DKK1	5.000	6253±3232
zVAD-fmk	5.000	2514±833
DKK1+zVAD-fmk	5.000	13311±1478

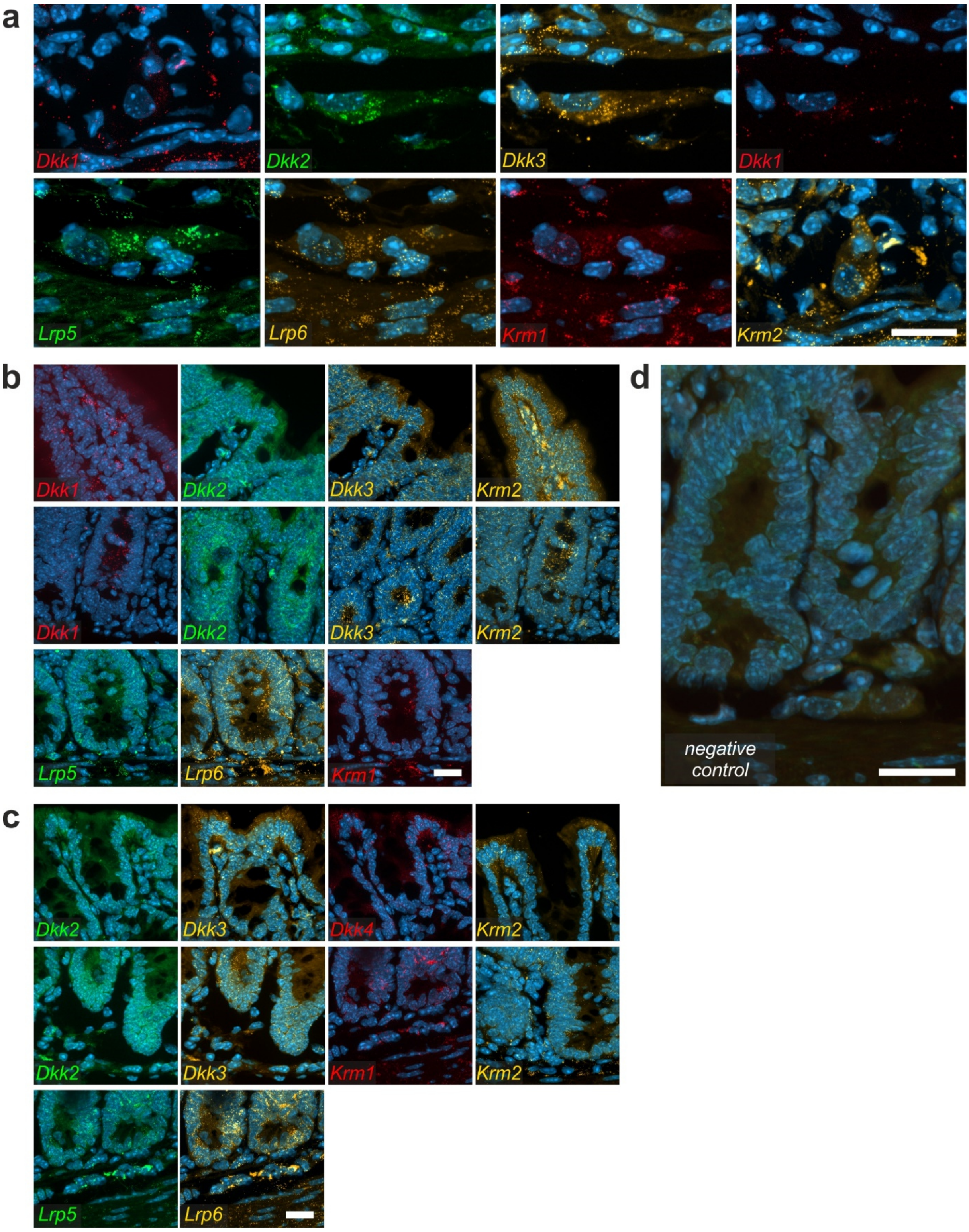

Suppl. Fig. 1: mRNA expression of DKK-ligands and -receptors within *Tunica mucosa* and *Tunica muscularis* of murine small and large intestine. Representative micrographs show DKK-ligands and corresponding receptor mRNA expression as indicated as well as nuclei staining with DAPI (blue) within submucosal ganglia **(a)** and within the *Tunica mucosa* **(b)** of the murine small intestine. **Scale bar: 20 µm. c:** displays the expression of DKK-ligands and corresponding receptors (as

indicated) as well as nuclei staining with DAPI (blue) within the *Tunica mucosa* of the murine large intestine. **Scale bar: 20 µm. d:** shows a representative negative control of murine large intestine. **Scale bar: 20 µm.**

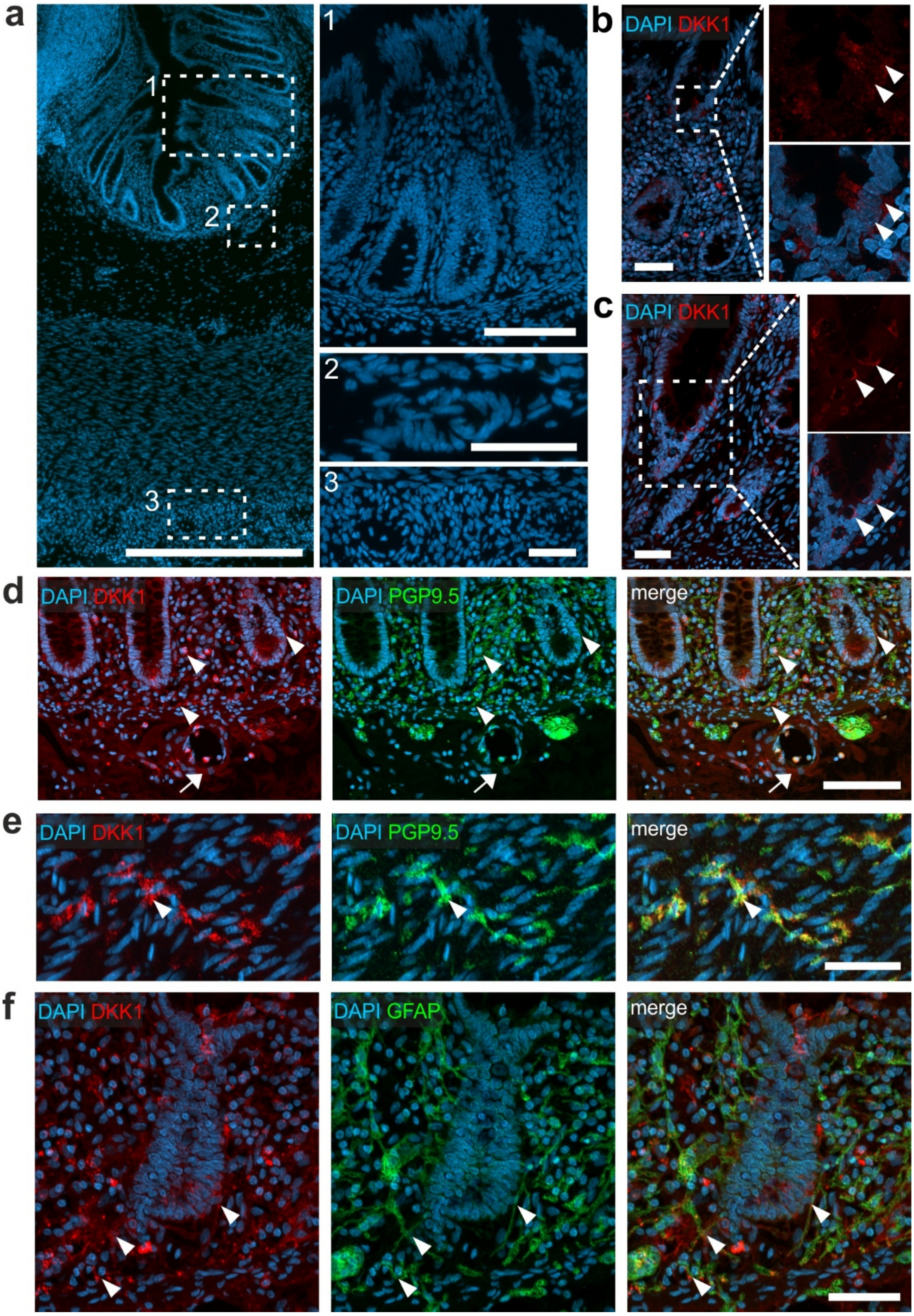

Suppl. Fig. 2: DKK1-expression in the *Tunica mucosa* and *Tunica muscularis* of human small and large intestine. a: displays an overview of a transversal section of the human colon stained with secondary antibodies only as a negative control and the nuclear stain DAPI (blue). White rectangles indicate the location of the high-power magnification micrographs 1-3 on the right. **Scale bars:** overview **500 µm**; details: **100**

µm (1), (2) and **50 µm** (3). **b and c:** show representative images of the *Tunica mucosa* stained for DKK1 (red) and DAPI (blue). The white rectangles show high-power magnification of DKK1-stained cells located at the bottom of the crypt (arrow head). **Scale bar: 50 µm. d:** shows representative image of human small intestine stained for stained for DKK1 (red), PGP9.5 (green) and the nuclear stain DAPI (blue). Arrowheads indicate $DKK1^+$ and $PGP9.5^+$ enteric neurites within the *Tunica mucosa*. The arrow marks DKK1-negative blood vessel. **Scale bar: 100 µm. e:** shows DKK1 (red) co-labeling with PGP9.5 (green) fibers within the *Tunica muscularis* (arrow head) of the human small intestine **Scale bars: 50 µm. f:** displays representative image of human small intestine stained for DKK1 (red), GFAP (green) and the nuclear stain DAPI (blue). Arrowheads indicate $DKK1^+$ and $GFAP^+$ type-III enteric glial cells within the *Tunica mucosa*. **Scale bar: 50µm.**

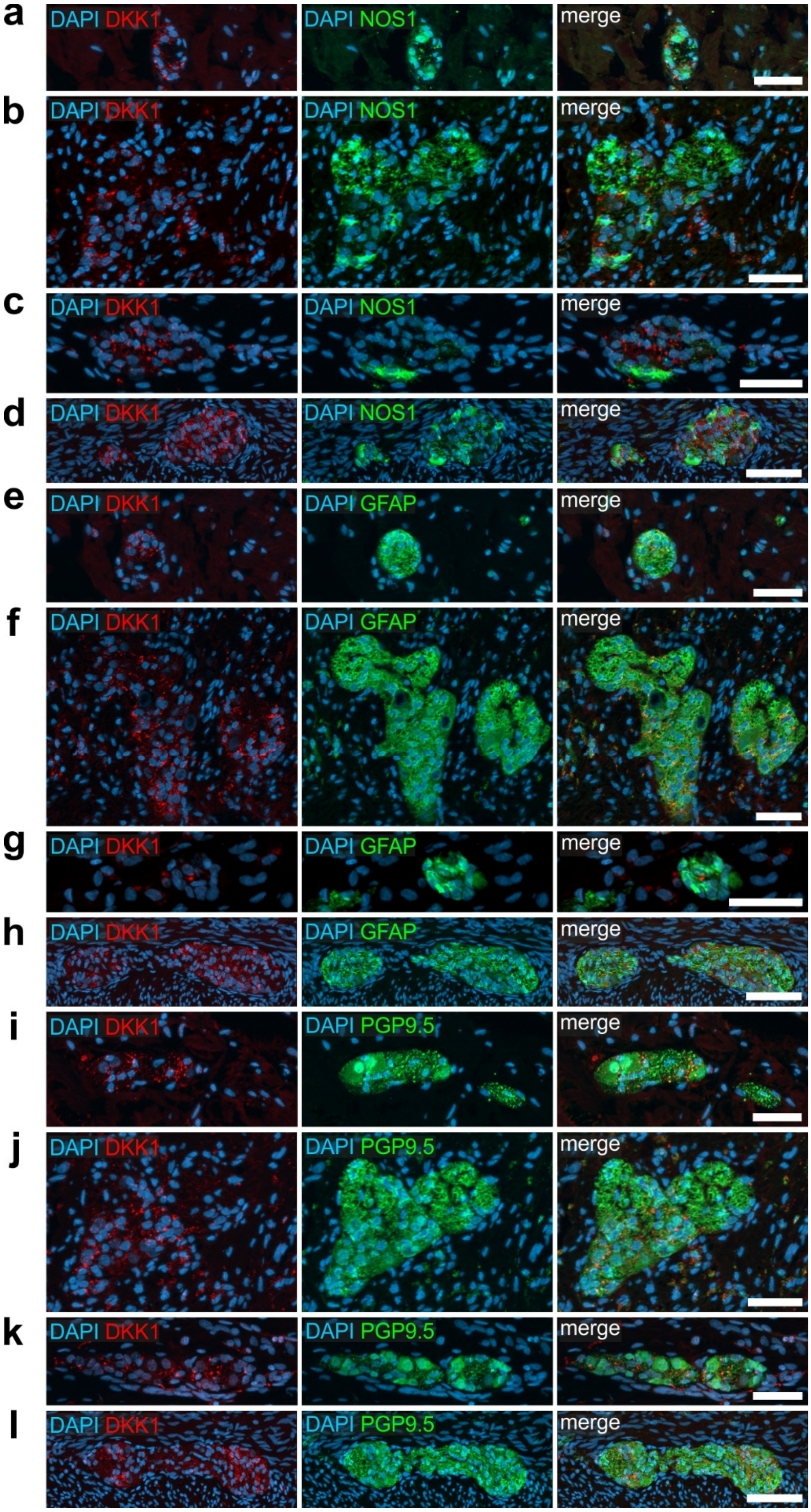

Suppl. Fig. 3: Co-labeling studies with DKK1 in human small and large intestine.
a, c: show a representative image of submucosal and, in **b, d**, a myenteric ganglion of small and large intestine respectively. Samples were stained for DKK1 (red), NOS1

(green), and nuclear marker DAPI (blue). **e, g:** display a representative image of submucosal and, in **f, h**, a myenteric ganglion of small and large intestine respectively. Samples were stained for DKK1 (red), GFAP (green), and with the nuclear marker DAPI (blue). **i, k:** show a representative submucosal and in, **j, l**, a myenteric ganglion of small and large intestine respectively. Samples were stained for DKK1 (red), the PGP9.5 (green), and with the nuclear marker DAPI (blue). **Scale bars: 50 µm**.

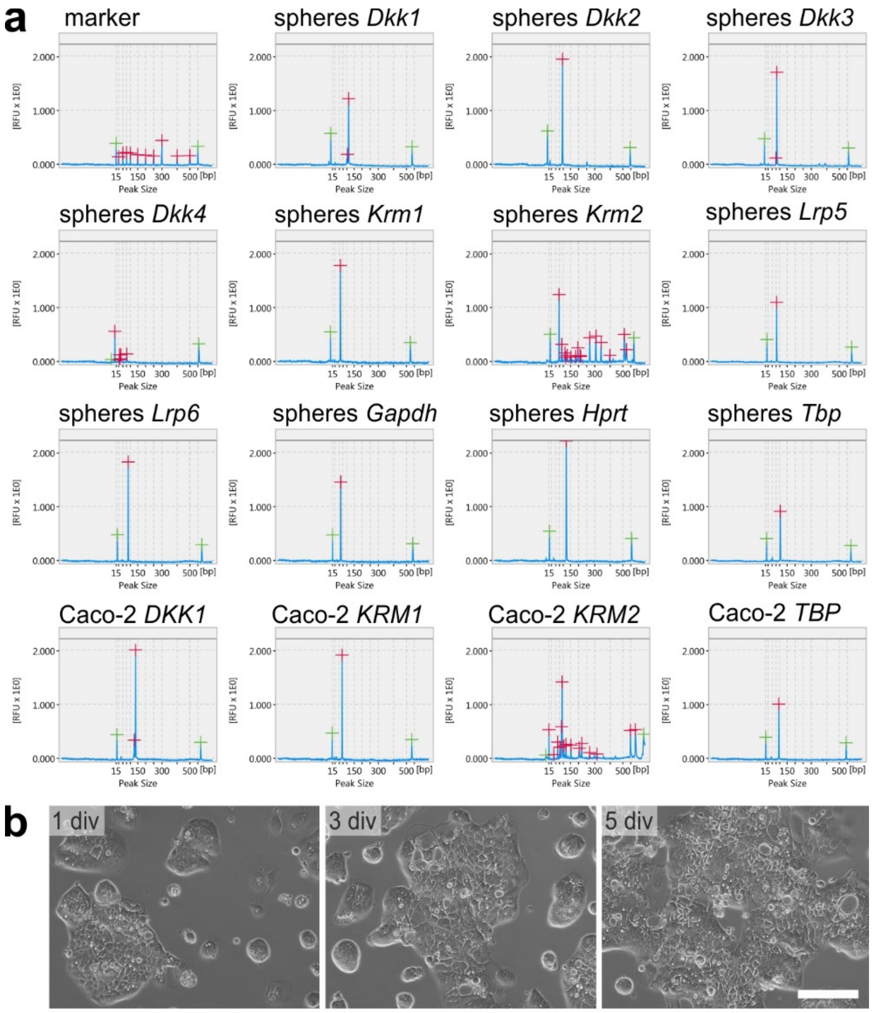

ligand receptor	mean CT value	± SD
DKK1	20.79	2.16
KRM1	30.60	0.14
KRM2	undetectable	
TBP	28.15	2.03

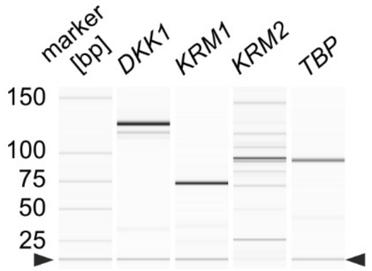

Suppl. Fig. 4: Electropherograms of DKK-ligand and -receptor detection. a: show representative electropherograms of DKK-ligands and -receptors expressed in enterospheres as well as in Caco-2 cells, which served as positive controls. Each blot indicates the size range in base pairs (bp) and the intensity of detected cDNA fragments. Within each blot the red cross represents the peak size that could be detected within the detection range marked by the alignment marker (green crosses). **b:** Micrographs show representative culture and morphology of Caco-2 cells (passage 29) after 5 days *in-vitro* (5 div). **Scale bar: 50 µm**. Lower left panel shows CT values of *DKK1*, the DKK-receptor *KRM1*, and housekeeping gene *TBP* identified by RT-PCR

in Caco-2 cells (passage 29) after 5 div. Lower right panel shows corresponding capillary gel image (arrow head indicates position of alignment marker).

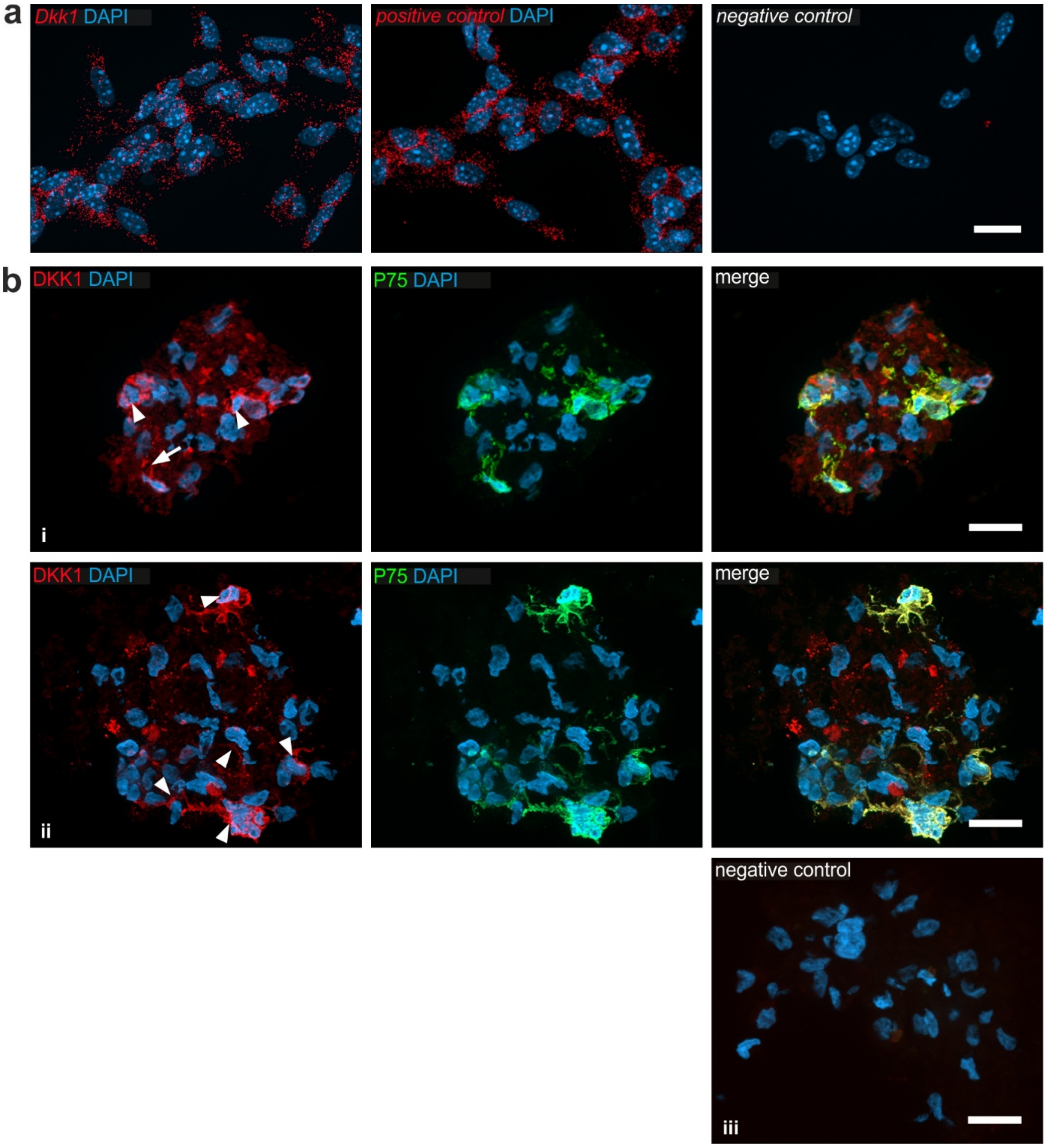

Suppl. Fig. 5: mRNA expression and protein expression of DKK1 in proliferating murine and human cell cultures. a: shows a representative image of *Dkk1*-expression (red) and with the nuclear marker DAPI (blue) in purified ENS-progenitor cells after 5 days *in vitro* analyzed by *in situ* hybridization experiments. **b:** depicts two representative images of kryosections from human spheres after 14 days under proliferative conditions (**b-i and b-ii**) stained for DKK1 (red), P75 (green) and the nuclear marker DAPI (blue). Arrowheads indicate DKK1^{+} and P75^{+} cells and the arrow marks DKK1^{+} and P75^{+} fibers. **b-iii** displays a human enterospheres stained with

secondary antibodies only as a negative control and the nuclear stain DAPI (blue). **Scale bars: 20 µm.**

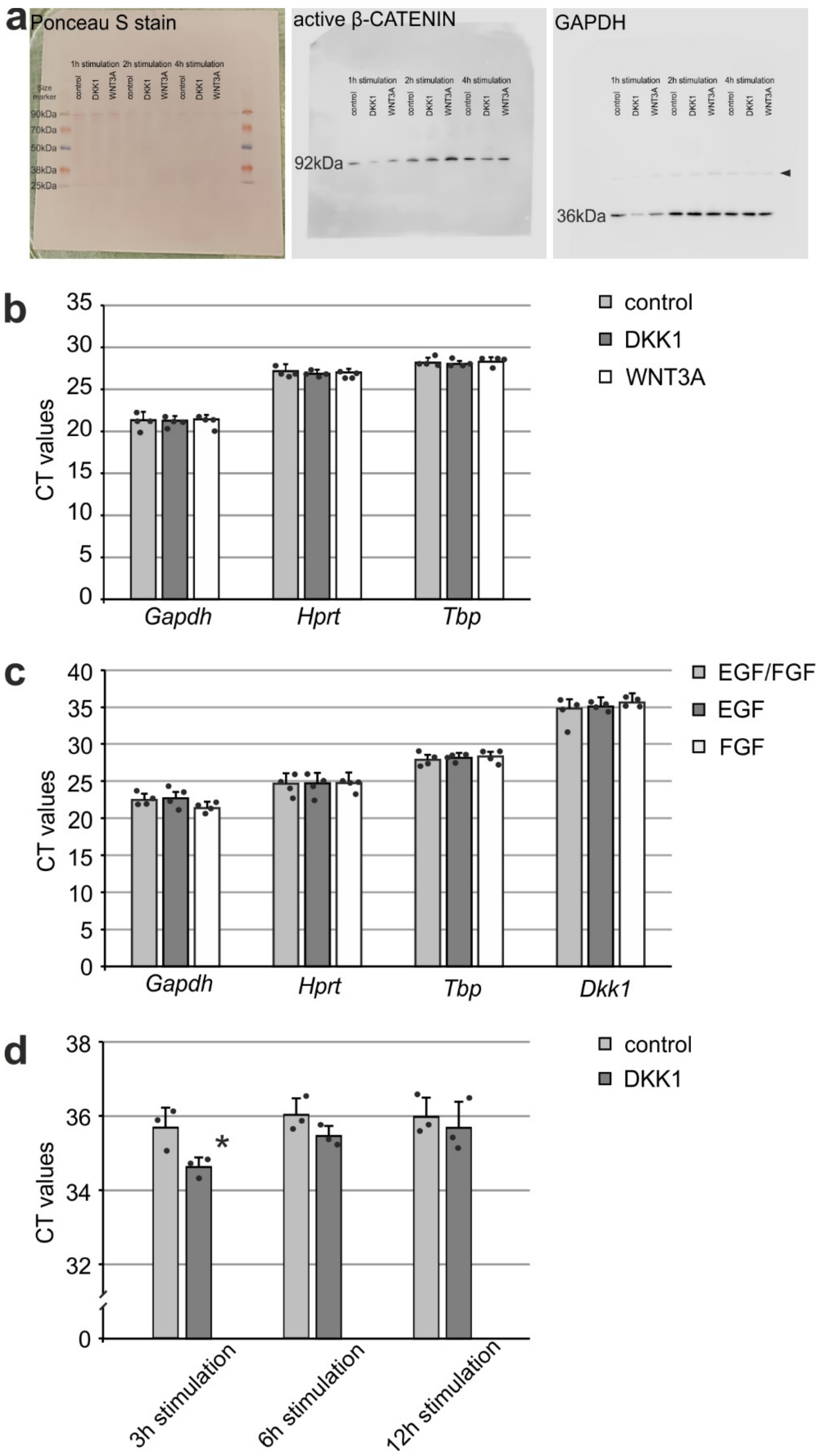

Suppl. Fig 6: Gene- and protein-expression analyses for DKK1 experiments. a: Time-course of active-β-CATENIN and GAPDH protein expression after DKK1-stimulation in murine proliferating enterospheres. Whole Western-Blot images of one representative Western-Blot experiment shows active β-CATENIN and GAPDH detection for the control, DKK1- and WNT3A-treated group after 1 hour, 2 hours and 4 hours of stimulation on the same blot. Protein bands for active-β-CATENIN with 92kDa

and GAPDH with 36kDa size can be clearly separated (see also weak band for active-β-CATENIN after following GAPDH detection, arrow head) **b:** DKK1- or WNT3A-stimulation did not alter the housekeeping-gene expression in proliferating murine enterospheres. The housekeeping genes Gapdh, Hprt and Tbp used for qRT-PCR experiments were regulated neither by DKK1 nor WNT3A treatment in enterospheres after 5 days in vitro (CT-values expressed as mean±SD, n=3). The dots represent individual data points. c: Cell culture conditions did not alter the housekeeping-gene expression of Gapdh, Hprt and Tbp as well as Dkk1-expression in enterospheres after 5 days in vitro (CT-values expressed as mean±SD, n=3). The dots represent individual data points d: Dkk1-expression was slightly upregulated in proliferating murine enterospheres after three hours following DKK1-stimulation (CT-values expressed as mean±SD, n=3). The asterisk indicates significant differences to control (ANOVA, Fisher LSD post-hoc test; n=3; 3h: DKK1 vs. control P=0.041, 6h: DKK1 vs. control P=0.142, 12h: DKK1 vs control P=0.592), the dots represent individual data points.

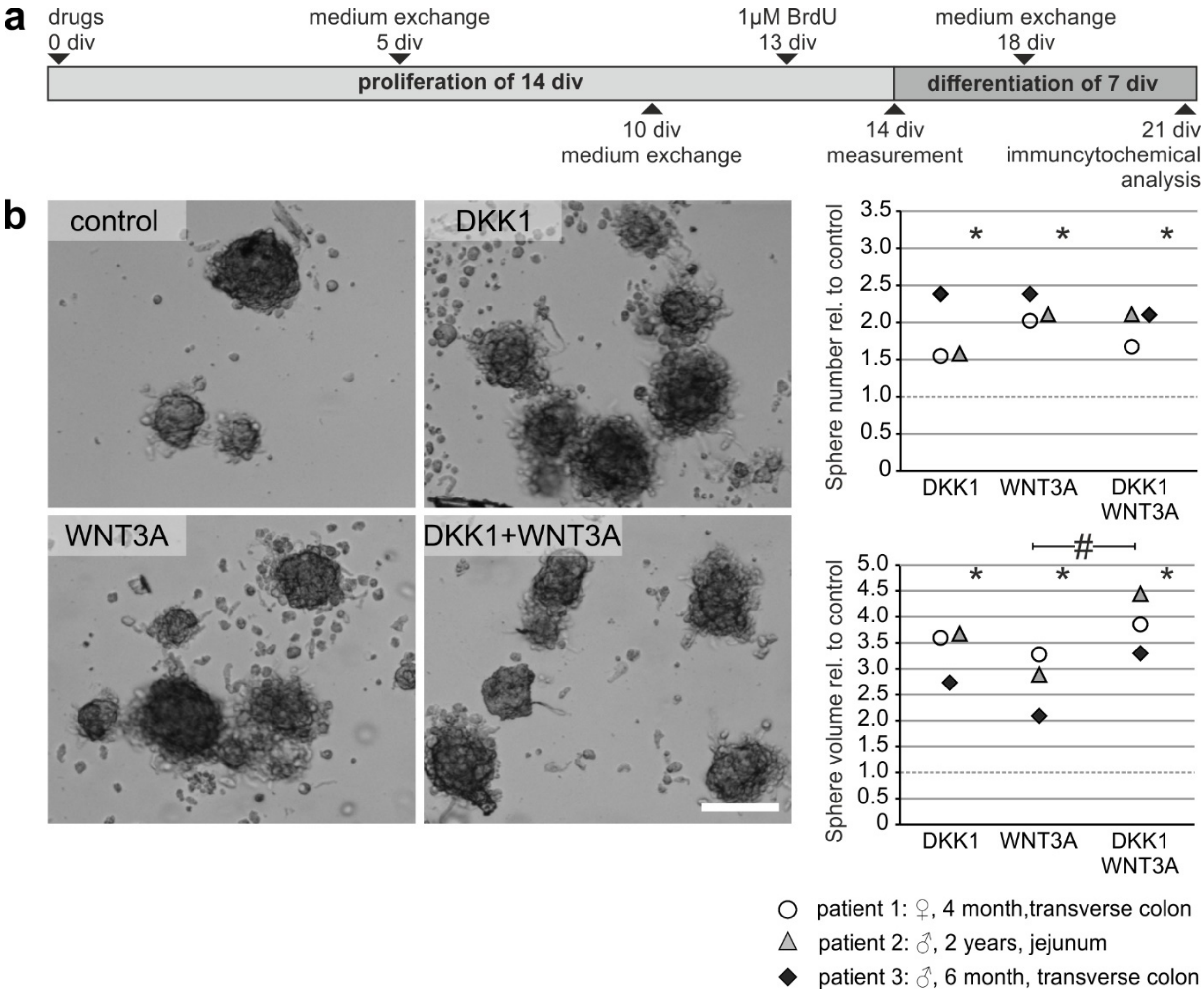

Suppl. Fig. 7: DKK1-stimulation increases the proliferation of human ENS-progenitors. a: The timeline demonstrates, that human ENS-progenitors were cultured for 14 days *in-vitro* (div) under proliferative conditions. DKK1 and/or WNT3A were applied directly upon seeding. **b:** Micrographs depict representative human enterospheres after 14 div under control, DKK1- and/or WNT3A-stimulation condition. **Scale bar: 50 µm.** The upper plot shows the number of enterosphere relative to control (mean±SD). Data points for 3 independent experiments (i.e., different patients) are represented by different symbols (in total 3324 human enterospheres were analyzed). Asterisk indicates significant differences compared to the control group (ANOVA followed by Fisher LSD post-hoc test; n=3; DKK1 vs. control: 0.020, WNT3A vs. control: 0.002, DKK1+WNT3A vs. control: 0.006). The plot below depicts, the cumulative volume of enterospheres for the control group and different treatments after 14 div is plotted. Data points for 3 independent experiments (i.e., different patients) are represented by different symbols (in total 3324 human enterospheres were analyzed). Asterisk indicates significant differences compared to the control group and the hash depicts significant difference between groups (ANOVA followed by Fisher LSD post-

hoc test for spheroid volume; n=3; DKK1 vs. control: 0.002, WNT3A vs. control: 0.006, DKK1+WNT3A vs. control: <0.001, DKK1+WNT3A vs. WNT3A: 0.026).

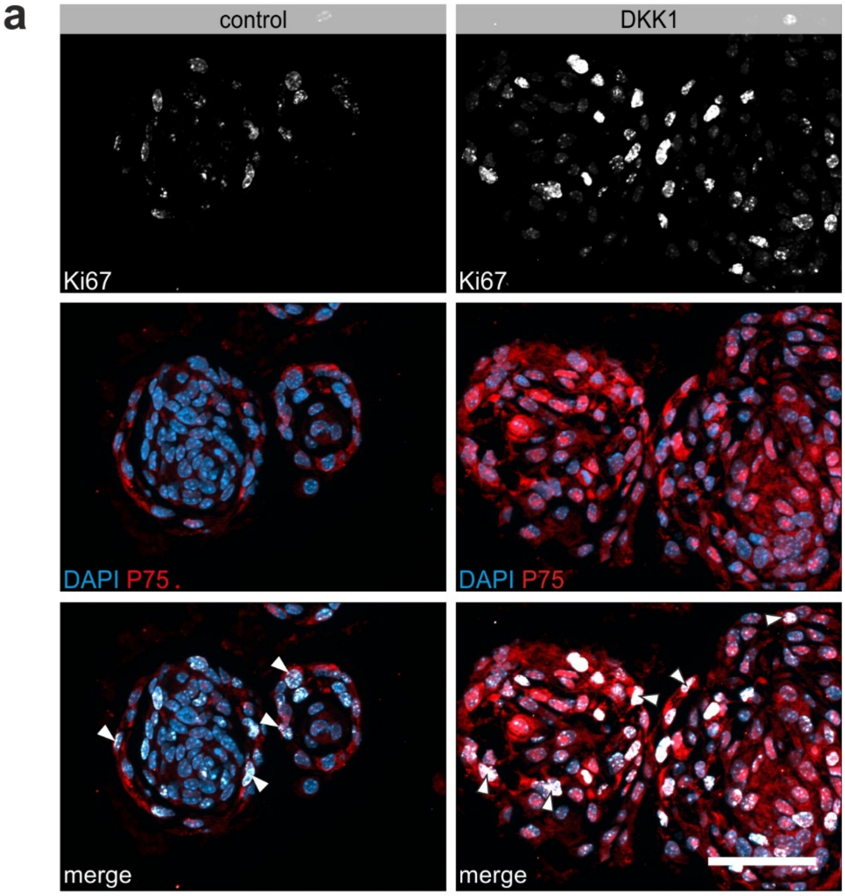

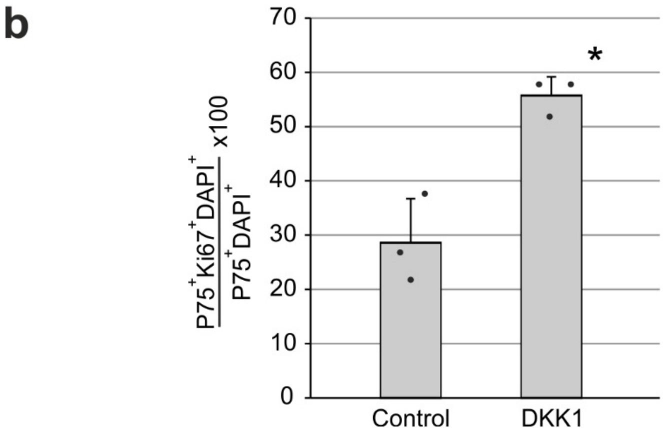

Suppl. Fig 8: DKK1-stimulus increased the amount of $P75^+Ki67^+$ cells in proliferating murine enterospheres. a: Micrographs display immunfluoresence co-labeling studies with Ki67 (white) and P75 (red) and the nuclear marker DAPI (blue), on paraffin-sections of enterospheres after 5 days *in vitro* for the control and DKK1-treated group. Arrowheads mark exemplary $Ki67^+$ and $P75^+$ cells. **Scale bar: 20 µm. b:** The barplots indicate the percentage of $P75^+Ki67^+$ cells (mean±SD) for the control and DKK1-stimulated group. DKK1-stimulation increased the number of $P75^+Ki67^+$ cells. The asterisk indicates significant differences compared to control (Student t-test; n=3; P=0.006), the dots represent individual data points.

a

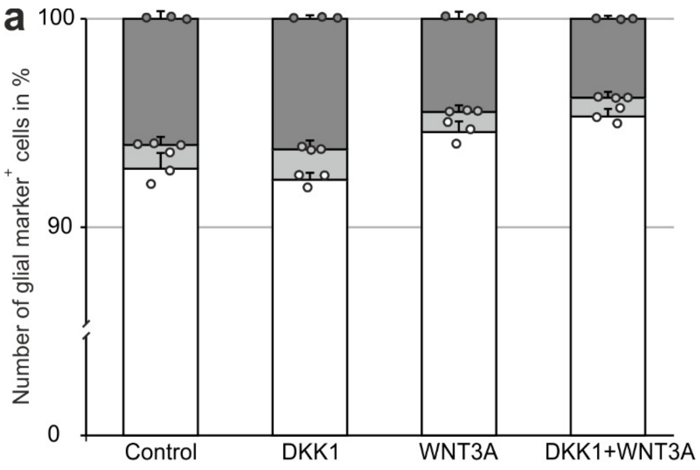

	Control	DKK1	WNT3A	DKK1+WNT3A
Sox10$^+$	6.05±0.37	6.27±0.17	4.47±0.35	3.78±0.15
S100β$^+$	1.14±0.39	1.45±0.44	0.97±0.32	0.91±0.29
Sox10$^+$S100β$^+$	92.8±0.76	92.2±1.45	94.6±0.53	95.3±0.37

b

Sox10$^+$S100β$^+$

**group vs group**	**P value**
WNT3A vs. control	0.004
DKK1+WNT3A vs. control	<0.001
DKK1 vs. WNT3A	<0.001
DKK1 vs. DKK1+WNT3A	<0.001

Sox10$^+$

**group vs group**	**P value**
DKK1 vs. control	<0.001
WNT3A vs. control	0.372
DKK1+WNT3A vs. control	<0.001
DKK1 vs. WNT3A	<0.001
DKK1 vs. DKK1+WNT3A	0.017
DKK1+WNT3A vs. WNT3A	<0.001

S100β$^+$

**group vs group**	**P value**
no significant difference	0.332

Suppl. Fig. 9: Differential expression of enteric glial cell marker SOX10 and S100β *in-vitro*. Bargraph displays the quantification of the percentage of SOX10$^+$ and/or S100 beta$^+$ enteric glial cells (mean±SD) of all cells expressing at least one glial marker for control, DKK1, WNT3A and combinatory group of 3 independent experiments. The dots represent individual data points for each subgroup. The lower panel provides the corresponding data expressed as mean±SD in percent (see also Supplementary Table 7 for total counts).

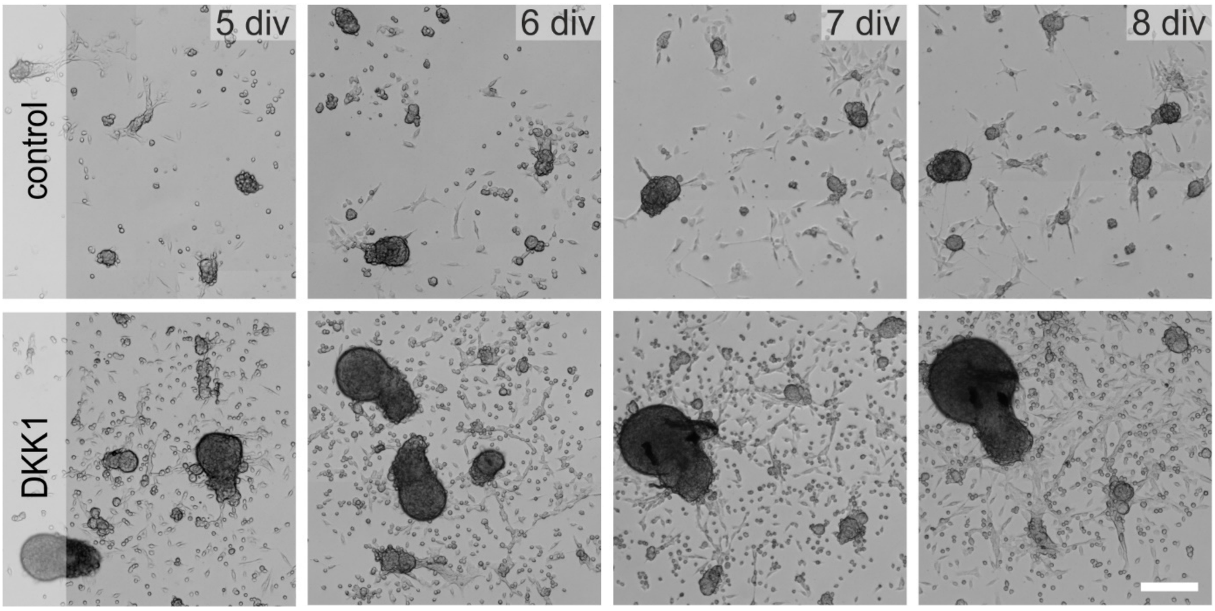

Suppl. Fig. 10: Long-term proliferation of murine enteropsheres under DKK1-stimulation. Brightfield images depict cultured murine enterospheres under proliferative conditions for 5 to 8 days *in vitro*. DKK1-stimulated spheres tend to fall apart into smaller fragments as well as single cells, whereas control-spheres maintain their spheroid shape within 8 div. **Scale bar: 200 µm.**

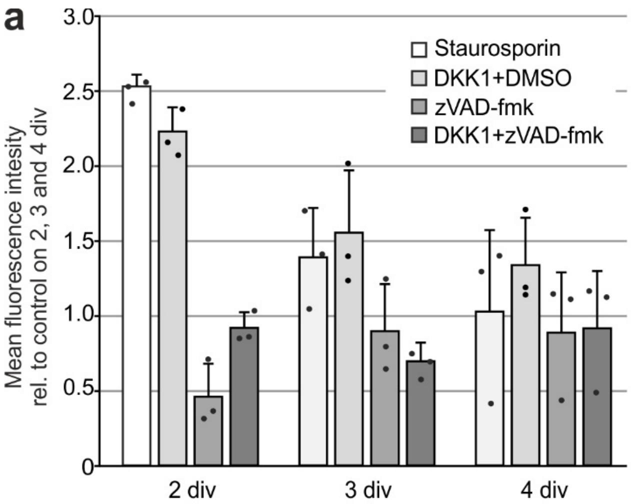

b

2 div

**group vs group**	**P value**
DKK1 vs. control	<0.001
zVAD-fmk vs. control	0.006
DKK1+zVAD-fmk vs. control	<0.001
DKK1 vs. zVAD-fmk	<0.001
DKK1 vs. DKK1+zVAD-fmk	<0.001
DKK1+zVAD-fmk vs. zVAD-fmk	0.020

3 div

**group vs group**	**P value**
DKK1 vs. control	0.038
DKK1 vs. zVAD-fmk	0.017
DKK1 vs. DKK1+zVAD-fmk	0.039

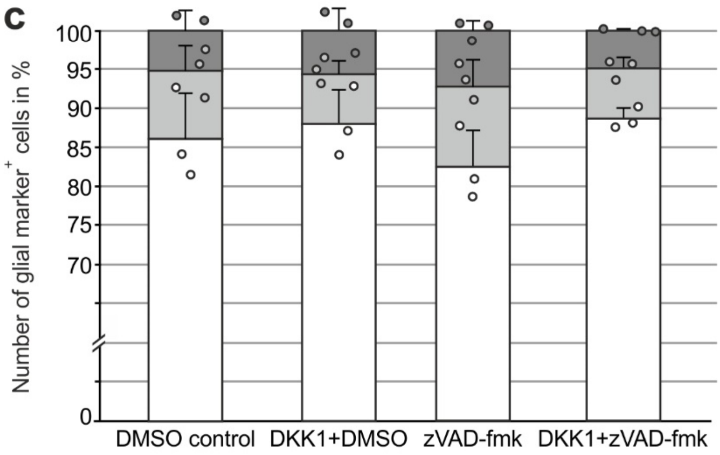

	DMSO control	DKK1+DMSO	zVAD-fmk	DKK1+zVAD-fmk
Sox10$^+$	5.25±2.62	5.65±2.75	7.31±1.26	4.93±0.17
S100β$^+$	8.70±3.32	6.41±1.69	10.3±3.46	6.46±1.29
Sox10$^+$S100β$^+$	86.1±5.83	87.9±4.42	82.5±4.70	88.6±1.38

d

SOX10$^+$S100β$^+$

**group vs group**	**P value**
no significant difference	0.367

SOX10$^+$

**group vs group**	**P value**
no significant difference	0.515

S100β$^+$

**group vs group**	**P value**
no significant difference	0.275

Suppl. Fig. 11: DKK1-mediated cell-death in proliferating murine ENS-progenitors can be rescued by the pan-Caspase-inhibitor zVAD-fmk. a: shows the fold change of mean fluorescence intensity (Caspase 3/7 activity), detected within four days of culture. Data shown was gathered from 3 independent experiments (mean±SD), in which cells treated with Staurosporine served as positive control. **b:** provides the statistics for group-wise comparisons. **c:** demonstrates the quantification of SOX10$^+$ and/or S100 beta$^+$ enteric glial cells in percent of all cells expressing at least one glial marker counted as indicated for the DMSO-control, DKK1+DMSO, zVAD-fmk and the combinatory-treated group. These data are expressed as mean±SD in percent. The lower panel provides the corresponding data expressed as mean-±SD in percent (see also Supplementary Table 7 for total counts). **d:** summarizes the statistics for group-wise comparisons.

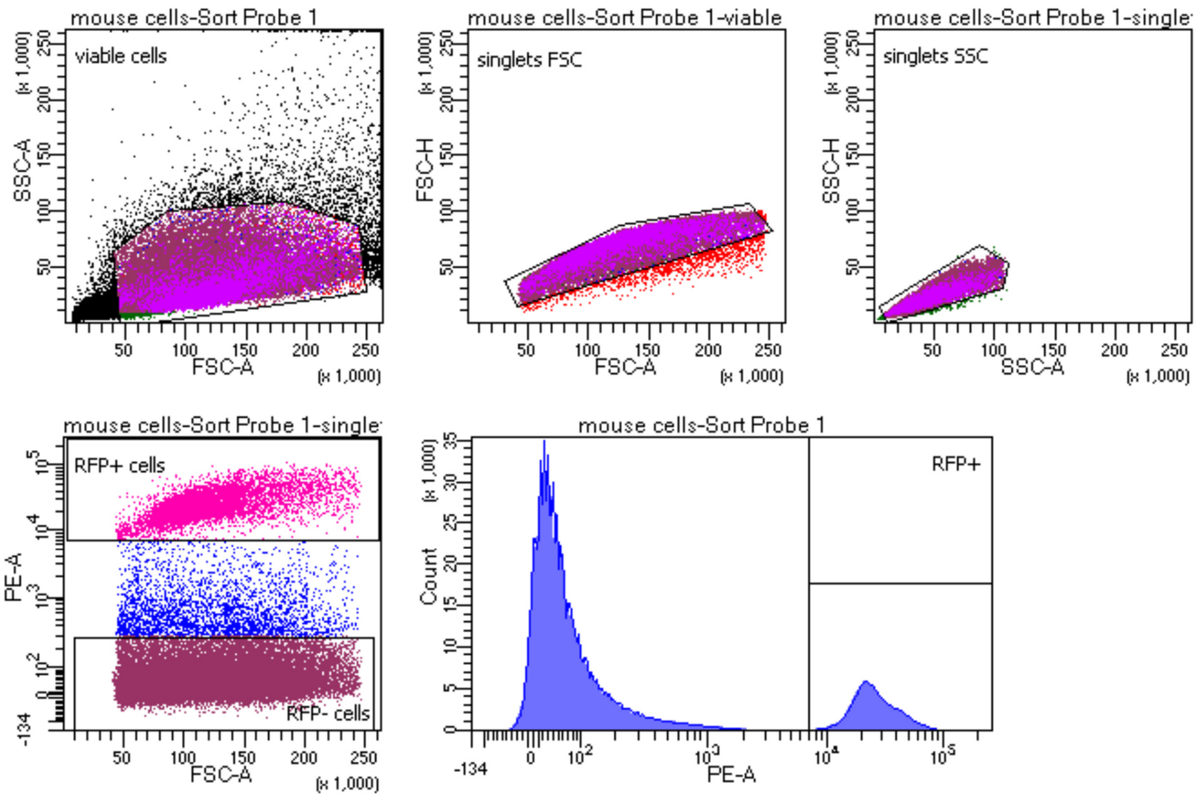

Tube: Sort Probe 1

Population	#Events	%Parent	%Total
All Events	1,279,965	####	100.0
viable cells	951,824	74.4	74.4
singlets FSC	925,621	97.2	72.3
singlets SSC	914,091	98.8	71.4
RFP+ cells	150,899	16.5	11.8
RFP+	150,922	16.5	11.8
RFP- cells	707,177	77.4	55.2

Suppl. Fig. 12: Exemplary gating protocol. Shown is the gating strategy used for the FACS-isolation of tdTomato-expressing (RFP+) ENS cells from one exemplary Tunica muscularis preparations of wnt1-tomato reporter mice.

Supplementary Material and Methods

Animals

Animals were handled and kept in accordance with the guidelines, regulating the handling of animals for scientific purposes (TierSchG paragraph 4, section 3, Notification number AT 01/19 M), which conform international guidelines. C57BL/6J were housed in standard cages with standard pathogen-free breeding and a standard 12-hour light/dark cycle at 22 ± 2 °C and 60 ± 5 % humidity. Germ-free food and water were available ad libitum. For isolation of enteric neuronal progenitor cells, neonatal (postnatal day 0) C57BL/6J mice were used without regard to sex. For *in situ* hybridization, small and large intestine samples from male C57BL/6J mice (postnatal day 60) were used.

For FACS-based isolation of ENS cells, we crossbred B6;129S6-Gt(ROSA)26Sortm9(CAG-tdTomato)Hze/J (Jackson Laboratory, Bar Harbor, ME; stock no. 007914) mice with B6.Cg-Tg(Wnt1-cre)2Sor/J (Jackson Laboratory; stock no. 022501) mice. The F1 offspring expressed tdTomato within all ENS cells and were used for experiments at postnatal day 0-5 of both sexes. These are termed wnt1-tomato in this work.

Human Specimens

Human gut samples were obtained from nine male and female patients aged 3 months to 3 years who were operated due to imperforate anus, intestinal obstruction syndrome, or short-gut syndrome (Supplementary Table1). All samples were collected after approval of the local ethical committee (Project Nr. 652/2019BO2) and with the consent of the patients parents according to the declaration of Helsinki.

Cell isolation of murine enteric neuronal progenitors

Neonate mice were euthanized in accordance with the guidelines of the German law (TierVersV). Thereby neonate mice were killed by decapitation. The whole intestine convolute was removed and transferred in murine preparation buffer (HBSS without Ca^{2+} / Mg^{2+} (Sigma-Aldrich, Taufkirchen, Germany), penicillin (100 U/mL; PAA, Cambridge,UK), streptomycin (100mg/mL; Sigma-Aldrich, Taufkirchen, Germany)). Adherent mesenteria were dissected, and the longitudinal and circular muscle layers containing myenteric plexus were stripped off the small intestine and collected in murine preperation buffer. After chopping, tissue enzymatically digested in a collagenase type XI (750 U/mL; Sigma-Aldrich, Taufkirchen, Germany)/dispase type II (250 mg/mL; Roche Diagnostics, Mannheim, Germany) was dissolved in Hanks' balanced salt solution (HBSS) with Ca^{2+} / Mg^{2+} (Sigma-Aldrich, Taufkirchen, Germany) for 20 minutes at 37 °C. Tissue was carefully triturated every 10 minutes with a fire-polished 1-mL pipette tip. Before the first trituration step, cell suspension was treated with 0.05 % (w/v) DNase I (Sigma-Aldrich, Taufkirchen, Germany). After tissue dissociation, 10 % (v/v) fetal calf serum (Biochrom, Berlin, Germany) was added and undigested larger tissue pieces were removed with a 30-µm cell strainer (Miltenyi Biotec GmbH, Bergisch Gladbach, Germany). To remove any residual enzymes, two washing steps in buffer (HBSS without Ca^{2+} / Mg^{2+} (Sigma-Aldrich, Taufkirchen, Germany), were performed at 200 g. The pellet was resuspended in murine proliferation media (Dulbecco's modified Eagle's medium with Ham's F12 medium (1:1; Life technologies, Darmstadt, Germany) containing N2 supplement (1:100; Life Technologies, Darmstadt, Germany), penicillin (100 U/mL; PAA, Cambridge,UK), streptomycin (100 mg/mL; Sigma-Aldrich, Taufkirchen, Germany), L-glutamine (2 mM; Sigma-Aldrich, Taufkirchen, Germany), EGF (20 ng/mL; Sigma-Aldrich, Taufkirchen,

Germany), and hbFGF (20 ng/mL; Sigma-Aldrich, Taufkirchen, Germany). Cells were seeded at a concentration of 2.0x10^4 cells/cm^2 and the media was supplemented with B27 (1:50; gibco® Thermo Fisher Scientific, MA, USA) once before seeding. Cells were cultured up to 5 days *in-vitro* (div) under proliferation conditions, whereby growth factors (20 ng/ml hEGF/20 ng/ml hbFGF) were added daily.

For long-term culture experiments after DKK1-stimulation, cells were seeded at a concentration of 2.0x10^4 cells/cm^2 and the media was supplemented with B27 (1:50; gibco® Thermo Fisher Scientific, MA, USA) once before seeding. Cells were cultured up to 8 days *in-vitro* (div) under proliferation conditions, whereby growth factors (20 ng/ml hEGF/20 ng/ml hbFGF) were added daily.

Cell isolation of human enteric neuronal progenitor cells

For human specimen, the resectates were cut open along the longitudinal axis and rinsed twice with human preparation buffer (HBSS without Ca^{2+} / Mg^{2+} (Sigma-Aldrich, Taufkirchen, Germany), penicillin (100 U/mL; PAA, Cambridge, UK), streptomycin (100mg/mL; Sigma-Aldrich, Taufkirchen, Germany), Cibrofloxacin Kabi (5mm/mL; Fresenius Kabi, Bad Homburg, Germany), and metronidazole (50 mg/mL; B. Braun, Melsungen, Germany). The *Tunica adventitia* and scar tissue were removed and the *Tunica muscularis* was peeled off the *Tela submucosa*. *Tunica muscularis* preparations were then stored in human preparation medium at 4 °C overnight. On the next day, the *Tunica muscularis* was chopped multiple times (800 mm each) using a McIlwain tissue chopper (Mickle Laboratory Engineering Co, Guildford, UK). The pieces were enzymatically digested in collagenase type XI (750 U/mL; Sigma-Aldrich, Taufkirchen, Germany)/dispase type II (250 mg/mL; Roche Diagnostics, Mannheim, Germany)

dissolved in Hanks' balanced salt solution (HBSS) with Ca^{2+} / Mg^{2+} (Sigma-Aldrich, Taufkirchen, Germany) containing 0.05 % (w/v) DNase I (Sigma-Aldrich Taufkirchen, Germany) and incubated for up to 60 minutes at 37 °C.

Tissue was triturated every 10 minutes with a fire-polished 25-mL serologic pipette. After tissue dissociation, fetal calf serum (Biochrom, Berlin, Germany) was added to a concentration of 10 % (v/v). The cells were pelleted at 200 g and erythrocyte lysis was performed using RBC Lysis buffer (eBioscience, Frankfurt a.M., Germany). After a second centrifugation at 200 g, the pellet was resuspended in HBSS without Ca^{2+} / Mg^{2+} (Sigma-Aldrich, Taufkirchen, Germany) and filtered using 100-µm, 70-µm, and 30-µm cell strainers. Cells were pelleted again at 200 g and resuspended in human proliferation medium (Dulbecco's modified Eagle's medium with Ham's F12 medium (1:1; Life technologies, Darmstadt, Germany) containing N2 supplement (1:100; Life Technologies, Darmstadt, Germany), Cibrofloxacin Kabi (5mm/mL; Fresenius Kabi, Bad Homburg, Germany), penicillin (100 U/mL; PAA, Cambridge,UK), streptomycin (100 mg/mL; Sigma-Aldrich, Taufkirchen, Germany), L-glutamine (2 mM; Sigma-Aldrich, Taufkirchen, Germany), EGF (20 ng/mL; Sigma-Aldrich, Taufkirchen, Germany), and bFGF (20 ng/mL; Sigma-Aldrich, Taufkirchen, Germany). Cell suspension was then filtered using a 30-mm cell strainer and seeded in a concentration of $2.0x10^4$ cells/cm^2. The medium was supplemented with B27 (1:50; gibco® Thermo Fisher Scientific, MA, USA) once before seeding. Cells were cultured up to 14 days *in-vitro* (14 div) under proliferation conditions, whereby growth factors (20 ng/ml hEGF/20 ng/ml hbFGF) were added daily and culture medium was exchanged every 5 days.

Fluorescence-Activated Cell Sorting

For FACS analysis, cells were isolated from the tunica muscularis of wnt1-tomato mice using the same procedure described here for wild-type mice. After the 30-µm straining step, cells were collected in Hibernate A (gibco® Thermo Fisher Scientific, MA, USA) medium supplemented with N2 supplement (1:100; Life Technologies, Darmstadt, Germany), penicillin (100 U/mL; PAA, Cambridge,UK), streptomycin (100 mg/mL; Sigma-Aldrich, Taufkirchen, Germany), L-glutamine (2 mM; Sigma-Aldrich, Taufkirchen, Germany), EGF (20 ng/mL; Sigma-Aldrich, Taufkirchen, Germany), and bFGF (20 ng/mL; Sigma-Aldrich, Taufkirchen, Germany) and B27 (1:50; gibco® Thermo Fisher Scientific, MA, USA). FACS was then performed with a BD FACS Aria flow cytometer (BD Biosciences) using a 100-µm nozzle. Forward-sideward scatter dot plots were used to exclude debris and cell aggregates (see also Supplementary Figure 12). Endogenous tdTomato was excited by a 488-nm laser. Emission filter was 576/26 nm. Purified cells were seeded as outlined before at a concentration of $1.0x10^5$ cells/cm^2.

Stimulation of murine and human enteric progenitor cells

Cell culture medium was supplemented either with WNT3A (20 ng/ml, R&D Systems, Inc., MN, USA), DKK1 (500 ng/ml, R&D Systems, Inc., MN, USA) or the combination of both on 1 day *in-vitro* (1 div) for murine cultures or on isolation day (0 div) for human cultures. Untreated cells served as control group. To quantify the absolute number of proliferating enteropsheres derived from neonate mice and human specimens brightfield images were taken of stimulated and unstimulated enteropsheres at 5 div respectively for human cultures at 14 div.

After 5 div, respectively 14 div, cell cultures were treated with 10 % (v/v) FCS for 2 hours to facilitate attachment of enteropsheres to culture dishes. Afterwards, differentiation medium ((Dulbecco's modified Eagle's medium with Ham's F12 medium (1:1; Life technologies, Darmstadt, Germany) containing N2 supplement (1:100; Life Technologies, Darmstadt, Germany), penicillin (100 U/mL; PAA, Cambridge,UK), streptomycin (100 mg/mL; Sigma-Aldrich, Taufkirchen, Germany), L-glutamine (2 mM; Sigma-Aldrich, Taufkirchen, Germany), 2 % (v/v) FCS (Biochrom AG, Berlin Germany) and ascorbic acid-2-phosphate (200 mM; Sigma-Aldrich, Taufkirchen, Germany) was added and exchanged on 8 div for murine cultures and on 18 div for human cultures. After 12, respectively 21 days *in-vitro*, cell cultures were fixed for immunocytochemical analysis.

Immunocytochemistry for differentiation markers and BrdU proliferation assay

After differentiation phase, murine neonate wildtype and human cell cultures were fixed with 4 % phosphate buffered paraformaldehyde (Merck KGaA, Darmstadt, Germany) for 20 minutes at room temperature and subsequently rinsed three times for 5 minutes in phosphate-buffered saline. In order to avoid unspecific binding of antibodies and to permeabilize cells for the detection of intracellular proteins, cell cultures were pre-treated with blocking solution containing 4 % goat serum (Biochrom, Berlin, Germany), 0.1 % bovine serum albumin (Roth, Karlsruhe, Germany), and 0.1 % Triton® X-100 (Roth, Karlsruhe, Germany), for 30 minutes at room temperature. Afterwards, primary antibodies (Supplementary Table 2) diluted in PBS, 0.1 % bovine serum albumin (Roth, Karlsruhe, Germany), and 0.1 % Triton® X-100 (Roth, Karlsruhe, Germany), were added and incubated overnight at 4 °C. Afterwards, cell cultures were rinsed with PBS three times for 5 minutes. Fluorescent conjugated secondary antibodies (Supplementary Table2), diluted

in PBS containing 0.1 % bovine serum albumin (Roth, Karlsruhe, Germany), and 0.1 % Triton® X-100 (Roth, Karlsruhe, Germany), were used for detection of primary antibodies and were incubated for one hour, light protected, at room temperature. Nuclear staining was performed together with the secondary antibody, using 4',6-diamidino-2-phenylindolestain (DAPI 200 ng/ml, Roth, Karlsruhe, Germany).

For BrdU proliferation assay, cell cultures were pre-treated with 2 N HCl (Roth, Karlsruhe, Germany) in a humidity chamber for 30 minutes at 37 °C after the incubation of fluorescent conjugated secondary antibodies. Next, three washing steps were carried out, utilizing two times borax buffer (0.1 M (w/v) di-Natriumtetraborat-10-hydrat pH: 8.5, Merck, Darmstadt Germany) and one-time PBS for 5 minutes followed by incubation of primary BrdU antibody (Supplementary Table2), diluted in PBS containing 0.1 % bovine serum albumin (Roth, Karlsruhe, Germany), and 0.1 % Triton® X-100 (Roth, Karlsruhe, Germany), in a humidity-chamber for 2 hours at 37 °C. Upon three washing steps with PBS for 5 minutes, cell cultures were treated with diluted secondary antibody in PBS containing 0.1 % bovine serum albumin (Roth, Karlsruhe, Germany), and 0.1 % Triton® X-100 (Roth, Karlsruhe, Germany), together with DAPI (200 ng/ml, Roth, Karlsruhe, Germany) for 1 hour at room temperature. Conclusively, cell cultures were rinsed again three times with PBS for 5 minutes.

Immunhistochemistry

Before embedding, tissue samples were fixed with 4 % phosphate buffered p-formaldehyde (Merck KGaA, Darmstadt, Germany) overnight at 4 °C and rinsed three times with phosphate-buffered saline (PBS).

For cryoconservation, fixed samples were stored overnight in 30 % sucrose solution (Applichem, Darmstadt, Germany) at 4 °C. Afterwards, samples were frozen in isopentane-nitrogen cooled TissueTek® (Sakura, Staufen, Germany) and stored at -80 °C until further processing. Before staining, cryosections (15 µm) were dried for one hour at room temperature, following rehydration with distilled water for 30 minutes.

For paraffin embedding, fixed tissue samples were dehydrated in an ascending alcohol series, followed by xylene and overnight infiltration of Paraffin at 60 °C. Before staining, paraffin sections (5 µm) were dewaxed by xylene and a descending alcohol series and were rinsed once with distilled water. Next, sections were pre-treated with boiled citric acid monohydrate buffer (10 mM, pH 6.0, Merck, Darmstadt, Germany) for three minutes and cooled down at room temperature.

To prevent unspecific binding of antibodies, samples were blocked for 30 minutes with PBS containing 4 % goat serum (Biochrom, Berlin, Germany), 0.1 % bovine serum albumin (Roth, Karlsruhe, Germany), and 0.1 % Triton® X-100 (Roth, Karlsruhe, Germany), followed by incubation of primary antibodies (Supplementary Table2) diluted in PBS with 0.1 % bovine serum albumin and 0.1 % Triton® X-100 overnight at 4 °C in a humidity chamber. Afterwards, samples were washed with PBS three times for 5 to 10 minutes. The secondary antibody (Supplementary Table2) was diluted in PBS, 0.1 % Triton X-100, and 0.1 % BSA and incubated for 60 minutes at room temperature. Nuclear

staining was carried out with 4′,6-diamidino-2-phenylindole (DAPI), (200 ng/ml, Roth, Karlsruhe, Germany). After two washing steps with PBS for 5 minutes, the samples were washed in distilled water for 5 minutes, followed by mounting with Kaiser's glycerol gelatine (Merck, Darmstadt, Germany).

In situ hybridization: Fixed frozen tissue sample preparation, pretreatment, RNAscope® HiPlex Assay and detection

We applied the RNAscope® HiPlex Assay and HiPlex Assay v2 (ACB biotechne, Wiesbaden-Nordenstadt Germany) according to the manufacturer's description to detect expression of relevant Dkk ligand and receptor mRNA in small and large intestine samples obtained from C57BL/6 mice (P60).

Sample preparation

The intestine bundle was removed from the abdomen, transferred and rapidly dissected in 4 % phosphate buffered p-formaldehyde (Merck KGaA, Darmstadt, Germany). Adherent mesenteria were dissected to unfold intestine bundle. Afterwards, small and large intestine were separated and were cut to size of approximately 2 cm samples.

Fixed frozen tissue sample preparation and pre-treatment

Before embedding, tissue samples were fixed with 4 % phosphate buffered p-formaldehyde (Merck KGaA, Darmstadt, Germany) for one hour at 4 °C and rinsed 3 times with RNAse-free phosphate-buffered saline (PBS). Afterwards, fixed samples were stored in 30 % sucrose solution (AppliChem, Darmstadt, Germany) over night at 4 °C. Afterwards, samples were frozen in isopentane-nitrogen cooled TissueTek® (Sakura, Staufen, Germany) and stored at -80 °C until further processing. Prepared cryosections

(12 µm) were washed with PBS and dried for 30 minutes at 60 °C, following post-fixation with 4 % phosphate buffered p-formaldehyde for 15 minutes at 4 °C. Before antigen retrieval, sections were dehydrated each time for 5 minutes in 50 % - 70 % and two times 100 % EtOH at room temperature. Target retrieval was performed in mild-boiling (98 – 102 °C) 1x RNAscope® Target Retrieval Reagent solution for 5 minutes, following one washing steps in distilled water and one in 100 % EtOH. Sections were dried completely at 60 °C for 5 minutes before the hydrophobic barrier was drawn. One the next day RNAscope® Protease III treatment was applied for 5 minutes at 40 °C. Before continuing with the RNAscope® HiPlex Assay, sections were washed once with distilled water.

RNAscope® HiPlex Assay

Before probe hybridization, pre-warmed RNAscope® HiPlex 50x probe stocks were diluted with RNAscope® HiPlex diluent according to the manufacturer's description and cooled to room temperature prior to use. Probe hybridization was carried out for 2 hours at 40 °C. Afterwards, sections were washed two times with 1x RNAscope® Wash Buffer for 2 minutes at room temperature. Next, three amplification rounds were carried out, while sections were treated successively first with the RNAscope® HiPlex Amp1, second the RNAscope® HiPlex Amp2 and third with the RNAscope® HiPlex Amp3 for 30 minutes at 40 °C. After each amplification round, sections were washed two times with 1x RNAscope® Wash Buffer for 2 minutes at room temperature.

For detection, sections were treated 15 minutes with the corresponding RNAscope® HiPlex Fluor solution at 40 °C, washed two times with 1x RNAscope® Wash Buffer for 2 minutes at room temperature, counterstained with RNAscope® DAPI for 1 minute and mounted with ProLong Gold Antifade Mountant (Thermo Fisher Scientific, MA, USA). For

each experiment a positive and a negative control was performed to evaluate signal strength as well as background staining. Target probe signal was evaluated according to the manufacturer's description. Target probes and their corresponding probe channel are listed in Supplementary Table 4.

Ki67, PCNA stainings and Terminal Deoxynucleotidyltransferase–Mediated DeoxyuridineTriphosphate Nick-End Labeling assay

To assess proliferation and cell death within murine enteropsheres during the proliferation phase, 5-day-old enteropsheres were picked from the medium and fixed with 4 % phosphate buffered p-formaldehyde (Merck KGaA, Darmstadt, Germany) for 20 minutes and afterwards rinsed three times with Tris buffer (25 mmol/L Tris-HCl (Roth, Karlsruhe, Germany) pH 7.5). Supernatant was removed and enterospheres resuspended with 2 % agar (Roth, Karlsruhe, Germany) in Tris buffer. During solidification of the agar, enterospheres were accumulated at the cup bottom by centrifugation at 6000 g for 10 seconds. Agar cones were withdrawn from the tubes and embedded in paraffin. Before staining, paraffin sections (5 µm) were dewaxed by xylene and a descending alcohol series and were rinsed once with distilled water. Next, sections were pre-treated with boiled citric acid buffer (10 mM, pH 6.0, Merck, Darmstadt, Germany) for three minutes and cooled down to room temperature. Afterwards, the immunocytochemical workup was the same as described above.

We also applied the Click-iT™ TUNEL Alexa Fluor™ 488 Imaging Assay (Invitrogen, USA) according to the manufacturer's description to detect DNA damage associated with apoptosis. As a positive control, we pre-treated sections with 0.005 % DNase I (Sigma-Aldrich, Taufkirchen, Germany) for 10 minutes at room temperature before terminal

deoxynucleotidyl transferase–mediated deoxyuridine triphosphate nick-end labeling staining. Sections treated with Click-iT® Reaction cocktail served as a technical negative control. For Ki67, PCNA immunocytochemistry and terminal deoxynucleotidyltransferase–mediated deoxyuridine triphosphate nick-endlabeling assays we used 4′,6-diamidino-2-phenylindole (DAPI), (200 ng/ml, Roth, Karlsruhe, Germany) for nuclei detection.

Caspase-3/7 activity assay

To profile apoptotic cell populations based on caspase-3/7 activity we used the NucView® 488 Caspase-3/7 Substrate (Biotium, CA, USA) according to the manufacturer's description. Therefore, cells were seeded at a concentration of $1x10^5$ cells/cm^2 on collagen type I coated cell culture plates and kept under proliferative conditions, whereby growth factors (20 ng/ml hEGF/20 ng/ml hbFGF) were added daily for 5 days. On 1 div, cells were washed once with proliferation medium and afterwards treated either with DKK1 (500 ng/ml), zVAD-fmk (pan-Caspase Inhibitor, 50 µM, Selleckchem, PA, USA) or the combination of both. DMSO (AppliChem, Darmstadt, Germany) treated cells served as control group and it was also applied to DKK1-treated group. 10 µM NucView® 488 Caspase-3/7 Substrate was added to each group. Cells treated with Staurosporine (1 µM, Selleckchem, PA, USA) served as positive control. Cells treated without NucView® 488 Caspase-3/7 Substrate served for background evaluation. On each day of cultivation, immunfluoresence images were taken to evaluate Caspase-3/7 activity over time.

Molecularbiology

RNA isolation and reverse transcriptase-PCR

Total RNA of enteropsheres was isolated using the RNeasy Plus Mini Kit (Qiagen, Hilden, Germany) according to manufacturer's instructions. RNA concentration and integrity were analysed using QIAxcel Advanced (Qiagen, Hilden, Germany) according to manufacturer's instructions. Reverse transcription was carried out with QuantiTect Reverse Transcription Kit (Qiagen, Hilden, Germany). Purified RNA treated without reverse transcriptase served as a negative control.

PCR was performed using the StepOnePlus™ Real-Time PCR System (Applied Biosystems, Darmstadt, Germany) and the PerfeCTa qPCR ToughMix ROX (Quantabio, Berverly, MA, USA) according to the manual instructions. The PCR conditions were 50 °C for 120 s and 95 °C for 10 min followed by 40 cycles of 95 °C for 15 s and 60 °C for 60 s. The acquisition was performed after the 60 °C step of each cycle. Glyceraldehyde 3-phosphate dehydrogenase (GAPDH), hypoxanthine-guanine phosphoribosyltrans-ferase (HPRT), and the TATA-binding protein (TBP) were used as reference genes. qRT-PCR was carried out following MIQE quality guidelines. Primers for ligand and receptors as well as the Wnt-target genes are shown in Supplementary Table 3.

Western blot analysis

Enteropsheres were picked and centrifuged at 200 g for 6 minutes. The pellet was rinsed twice with PBS, lysed using RIPA buffer (150 mM NaCl (Merck KGaA, Darmstadt, Germany); 5 mM EDTA (AppliChem, Darmstadt, Germany), pH 8.0; 10 mM TrismaBASE, pH 8.0 (Sigma-Aldrich, Taufkirchen, Germany); 1 % (v/v) Triton-X-100 (AppliChem, Darmstadt, Germany); 0.1 % (v/v) SDS (AppliChem, Darmstadt, Germany); 0.5 % (v/v) sodium deoxycholate (Sigma-Aldrich, Taufkirchen, Germany)). Protein concentrations were measured using the Qubit® Protein Assay Kit and the Qubit® Fluorometer (Life Technologies, Darmstadt, Germany). We used a methanol-chloroform method to precipitate the proteins and resuspended the pellet in sodium dodecylsulfate (SDS)–polyacrylamide gel electrophoresis (PAGE) sample buffer (3 % (v/v) SDS (AppliChem, Darmstadt, Germany), 10 mM TrismaBASE, pH 6.8 (Sigma-Aldrich, Taufkirchen, Germany), 6.0 % (v/v) glycerol (Sigma-Aldrich, Taufkirchen, Germany), 0.01 % (v/v) bromphenol blue (Sigma-Aldrich, Taufkirchen, Germany), and 0.1 M dithiothreitol (Roth)). Protein samples were protected from decay by adding protease inhibitor cocktail 5 MammCell/Tissue (1:100, AppliChem, Darmstadt, Germany). 40 µg of each protein sample were loaded onto a 4-12 % Bolt™ Bis-Tris Plus Mini Gel (Invitrogen, USA) and blotted onto a nitrocellulose membrane. Cameleon Duo pre-stained 8-260kDa was used as size marker (LI-COR Biosciences, NB, USA). Detection was carried out with the Pierce® Fast Western Blot Kit and the SuperSignal® West Femto Substrate (Thermo Fisher Scientific, MA, USA) using mouse anti-GAPDH antibody (Calbiochem, Darmstadt, Germany) and mouse anti-active β-catenin (Upstate, via Merck KGaA, Darmstadt, Germany). The blots of six independent experiments were scanned with the LICOR

Odyssey XF Imaging System (LI-COR Biosciences, NB, USA) with an exposure time of 10 minutes each. The optical density was analyzed using the software ImageJ.

Microscopy

Images were acquired using a Zeiss Axio Imager.Z1 fluorescence microscope with Apotome module with 358, 488, 543, 647 nm for excitation and appropriate filter sets. Images were acquired using ZEN software. For immuncyto- and histochemical stainings exposure time for DAPI was 200 ms, for DKK1 2000 ms and for the neuronal and glial markers 1000 ms with 20-objective. For *in situ* hybridization excitation time for DAPI was 150 ms and for the RNAscope HiPlex Fluoro solutions 5000 ms with 63-objective. For Caspase-3/7 activity assay excitation time was 1000 ms with 10-objective.

Data Analysis

For analysis of sphere growth, bright-field images were taken at time point 5 div for murine and 14 div for human cell cultivation. In total 1511 murine enteropspheres and 3324 human enteropsheres were analyzed. The area of spheroids was measured on bright-field images using Axiovision software (Zeiss, Oberkochen, Germany). Assuming an ideal sphere shape of enteropsheres, the theoretical diameter (d) was calculated by equation [1]: $d = 2x\sqrt{\frac{A}{\pi}}$ in µm whereby A is the measured area. Further, the total cell volume was calculated by equation [2]: $V = \frac{1}{6}\ x\ \pi d^3$ to evaluate a possible pharmacologically effect on the total cell volume of counted enteropsheres, whereby V is the obtained volume in µm³. It should be mentioned that human progenitors do not form compact spheroids with smooth edges as compared to mouse spheroids, for this reason we measured the dense compact core. For all, cumulative sphere number as well as volume was normalized to

controls to calculate fold change. For each group two technical replicates were analyzed. For the quantification of $Ki67^+$/$PCNA^+$/$P75^+$/$TUNEL^+$ cells, 500 DAPI positive nuclei with the respective co-labeling event was counted to calculate percentages. To quantify differentiated murine enteric neurons two technical replicates per experimental group were counted manually and mean values were calculated. For the quantification of murine SOX10 and S100beta positive cells one technical replicate was counted manually. For the analysis of human differentiation data three biological replicates per experimental group were manually counted and mean value was calculated. qRT-PCR experiments were carried out following MIQE quality guidelines with four independent experiments. For target gene expression under DKK1 or WNT3A stimulation, the $\Delta\Delta$ CT method was used to calculate the log-fold changes between the housekeeping gene and gene of interest. For Western Blot analysis, the relative density of the peaks between untreated and stimulated enteropsheres was calculated following the method outlined in the ImageJ documentation. For Caspase 3/7 activity assay, 30 random areas of interest (each 0.01 cm^2, total cultivated area $1cm^2$) were set and the mean fluorescence intensity was measured using the Axiovision software. Afterwards the mean intensity value was calculated, and background intensity was subtracted.
