# Supplementary material for: Dickkopf1 induces enteric neurogenesis and gliogenesis in vitro if apoptosis is evaded
Source: Commun Biol. 2023 Aug 2;6:808. doi: 10.1038/s42003-023-05072-x (PMC10397193; doi:10.1038/s42003-023-05072-x)
Supplement: Supplementary file 5 — Reporting Summary [file 42003_2023_5072_MOESM5_ESM.pdf]

nature portfolio

Corresponding author(s): Peter Neckel

Last updated by author(s): Jun 16, 2023

Reporting Summary

Nature Portfolio wishes to improve the reproducibility of the work that we publish. This form provides structure for consistency and transparency in reporting. For further information on Nature Portfolio policies, see our Editorial Policies and the Editorial Policy Checklist.

Statistics

For all statistical analyses, confirm that the following items are present in the figure legend, table legend, main text, or Methods section.

n/a	Confirmed	
☐	☒	The exact sample size (n) for each experimental group/condition, given as a discrete number and unit of measurement
☐	☒	A statement on whether measurements were taken from distinct samples or whether the same sample was measured repeatedly
☐	☒	The statistical test(s) used AND whether they are one- or two-sided<br>*Only common tests should be described solely by name; describe more complex techniques in the Methods section.*
☒	☐	A description of all covariates tested
☐	☒	A description of any assumptions or corrections, such as tests of normality and adjustment for multiple comparisons
☐	☒	A full description of the statistical parameters including central tendency (e.g. means) or other basic estimates (e.g. regression coefficient) AND variation (e.g. standard deviation) or associated estimates of uncertainty (e.g. confidence intervals)
☐	☒	For null hypothesis testing, the test statistic (e.g. F, t, r) with confidence intervals, effect sizes, degrees of freedom and P value noted<br>*Give P values as exact values whenever suitable.*
☒	☐	For Bayesian analysis, information on the choice of priors and Markov chain Monte Carlo settings
☒	☐	For hierarchical and complex designs, identification of the appropriate level for tests and full reporting of outcomes
☒	☐	Estimates of effect sizes (e.g. Cohen's d, Pearson's r), indicating how they were calculated

Our web collection on statistics for biologists contains articles on many of the points above.

Software and code

Policy information about availability of computer code

Data collection	ZeissZen blue, QIAxcel Advanced, StepOne Plus, LICOR odyssey XF Imaging System, Excel
Data analysis	Excel, SigmaStat, ImageJ, ZeissZen blue

For manuscripts utilizing custom algorithms or software that are central to the research but not yet described in published literature, software must be made available to editors and reviewers. We strongly encourage code deposition in a community repository (e.g. GitHub). See the Nature Portfolio guidelines for submitting code & software for further information.

Data

Policy information about availability of data

All manuscripts must include a data availability statement. This statement should provide the following information, where applicable:

- Accession codes, unique identifiers, or web links for publicly available datasets
- A description of any restrictions on data availability
- For clinical datasets or third party data, please ensure that the statement adheres to our policy

We add a data availability statement to the manuscript.

Research involving human participants, their data, or biological material

Policy information about studies with human participants or human data. See also policy information about sex, gender (identity/presentation), and sexual orientation and race, ethnicity and racism.

Reporting on sex and gender	Sex and gender was not considered, however, sex was determined and is provided in the Material&Method section.
Reporting on race, ethnicity, or other socially relevant groupings	Was not considered for this study.
Population characteristics	Relevant information, e.g., diagnosis is stated in the Material&Methods section.
Recruitment	All patients with surgical interventions on the intestinal tract at the Department of Pediatric Surgery, University Children's Hospital Tübingen, Germany.
Ethics oversight	University Tübingen, Ethics Committee at the Faculty of Medicine

Note that full information on the approval of the study protocol must also be provided in the manuscript.

Field-specific reporting

Please select the one below that is the best fit for your research. If you are not sure, read the appropriate sections before making your selection.

☒ Life sciences ☐ Behavioural & social sciences ☐ Ecological, evolutionary & environmental sciences

For a reference copy of the document with all sections, see nature.com/documents/nr-reporting-summary-flat.pdf

Life sciences study design

All studies must disclose on these points even when the disclosure is negative.

Sample size	For mouse and human data 3 samples were used.
Data exclusions	no data was excluded
Replication	For counting of differentiation data as well as measurements of spheroids two technical repilcates per group per experiment were conducted.
Randomization	per experiment 3 samples were collected from 3 individuals (mouse/human)
Blinding	Counting of differentiation data was done blinded.

Reporting for specific materials, systems and methods

We require information from authors about some types of materials, experimental systems and methods used in many studies. Here, indicate whether each material, system or method listed is relevant to your study. If you are not sure if a list item applies to your research, read the appropriate section before selecting a response.

Materials & experimental systems

n/a	Involved in the study	
☐	☒	Antibodies
☐	☒	Eukaryotic cell lines
☒	☐	Palaeontology and archaeology
☐	☒	Animals and other organisms
☒	☐	Clinical data
☒	☐	Dual use research of concern
☒	☐	Plants

Methods

n/a	Involved in the study	
☒	☐	ChIP-seq
☐	☒	Flow cytometry
☒	☐	MRI-based neuroimaging

Antibodies

Antibodies used	all antibodies used in this study are listed in the Material&Methods section.
Validation	Antibody validation by manufacturer and previous, independent publications

Eukaryotic cell lines

Policy information about cell lines and Sex and Gender in Research

Cell line source(s)	Provider: German Collection of Microorganisms and Cell Cultures GmbH, Caco-2 (DSMZ no.: ACC 169)
Authentication	Authentification by provider
Mycoplasma contamination	not testet after purchase from cell bank
Commonly misidentified lines (See ICLAC register)	n.a.

Animals and other research organisms

Policy information about studies involving animals; ARRIVE guidelines recommended for reporting animal research, and Sex and Gender in Research

Laboratory animals	C57BL/6J<br>B6;129S6-Gt(ROSA)26Sortm9(CAG-tdTomato)Hze/J (Jackson Laboratory, Bar Harbor, ME; stock no. 007914);<br>B6.Cg-Tg(Wnt1-cre)2Sor/J (Jackson Laboratory; stock no. 022501)
Wild animals	n.a.
Reporting on sex	Mice were used without regard to sex.
Field-collected samples	n.a.
Ethics oversight	handling of animals for scientific purposes (TierSchG paragraph 4, section 3, Notification number AT 01/19 M)

Note that full information on the approval of the study protocol must also be provided in the manuscript.

Flow Cytometry

Plots

Confirm that:

☒ The axis labels state the marker and fluorochrome used (e.g. CD4-FITC).

☒ The axis scales are clearly visible. Include numbers along axes only for bottom left plot of group (a 'group' is an analysis of identical markers).

☒ All plots are contour plots with outliers or pseudocolor plots.

☒ A numerical value for number of cells or percentage (with statistics) is provided.

Methodology

Sample preparation	Cell isolation procedure and further processing is described in the Material and Methods section.
Instrument	Cell Sorter ARIAIIIu (BG 501, 2nd F, R 581)
Software	BD FACSDiva 9.0.1
Cell population abundance	The RFP+ (tdTomato positive) sorted population makes up 11% from the total cell population.
Gating strategy	Forward-sideward-scatter dot plots were used to exclude debris and cell aggregates. Afterwards, cells were gated according to their tdTomato expression, wherby endogenous tdTomato was excited by a 488-nm laser. Emission filter was 576/26 nm.

☒ Tick this box to confirm that a figure exemplifying the gating strategy is provided in the Supplementary Information.
